# The Hyoscine Sleep in Obstetric Practice

**Published:** 1907-06

**Authors:** 


### SELECTIONS AND ABSTRACTS.

### THE HYOSCINE SLEEP IN OBSTETRIC PRACTICE.

Woodbridge Hall Birchmore believes that hyoscine is for the accoucheur the ideal anesthetic. He knows of no experience with an excessive dose. The importance of the hyoscine sleep in obstetric practice is amply demonstrated. It gives all the aid in quieting the patient that any narcotic can give, while in addition it affords a practical anesthesia of prolonged duration without risk to either mother or child. This anesthetic sleep is without danger. Hyoscine is a drug sedative to the cerebro-spinal axis, but not to the ganglia connected with the reflexes of common life.—Medical Record, January 17th, 1907.

